# Patient specific circulating tumor DNA fingerprints to monitor treatment response across multiple tumors

**DOI:** 10.1186/s12967-020-02449-y

**Published:** 2020-08-01

**Authors:** Jiaping Li, Wei Jiang, Jinwang Wei, Jianwei Zhang, Linbo Cai, Minjie Luo, Zhan Wang, Wending Sun, Shengzhou Wang, Chen Wang, Chun Dai, Jun Liu, Guan Wang, Jiping Wang, Qiang Xu, Yanhong Deng

**Affiliations:** 1 Department of Interventional Oncology, The First Affiliated Hospital of Sun Yat-sen University, Guangdong, China; 2 Department of Radiation Oncology, Huanhu Hospital, Tianjin, China; 3 GenomiCare Biotechnology Co. Ltd, Shanghai, China; 4 Department of Medical Oncology, The Sixth Affiliated Hospital of Sun Yat-sen University, No. 26 Erheng Road, Tianhe District, Guangzhou, 510655 China; 5 Department of Oncology, Guangdong 999 Brain Hospital, Guangdong, China; 6 Department of Pediatric Neurosurgery, Zhujiang Hospital of Southern Medical University, Guangdong, China; 7 Department of Medical Oncology, Changzheng Hospital, The Second Military Medical University, Shanghai, China; 8 Division of Surgical Oncology, Brigham and Women’s Hospital, Boston, MA USA

**Keywords:** Customized ctDNA panel, ctDNA fingerprint, Cancer treatment response, Whole exome sequencing, Drug-related mutation

## Abstract

**Background:**

Circulating tumor DNA (ctDNA) offers a convenient way to monitor tumor progression and treatment response. Because tumor mutational profiles are highly variable from person to person, a fixed content panel may be insufficient to track treatment response in all patients.

**Methods:**

We design ctDNA fingerprint panels specific to individual patients which are based on whole exome sequencing and target to high frequency clonal population clusters in patients. We test the fingerprint panels in 313 patients who together have eight tumor types (colorectal, hepatocellular, gastric, breast, pancreatic, and esophageal carcinomas and lung cancer and cholangiocarcinoma) and exposed to multiple treatment methods (surgery, chemotherapy, radiotherapy, targeted-drug therapy, immunotherapy, and combinations of them). We also monitor drug-related mutations in the patients using a pre-designed panel with eight hotspot genes.

**Results:**

291 (93.0%) designed fingerprint panels harbor less than ten previously known tumor genes. We detected 7475 ctDNA mutations in 238 (76%) patients and 6196 (96.0%) of the mutations are detected in only one test. Both the level of ctDNA content fraction (CCF) and fold change of CCF (between the definitive and proceeding tests) are highly correlated with clinical outcomes (p-values 1.36e-6 for level and 5.64e-10 for fold change, Kruskal–Wallis test). The CCFs of PD patients are an order of magnitude higher than the CCFs of SD and OR patients (median/mean 2.22%/8.96% for SD, 0.18/0.21% for PD, and 0.31/0.54% for OR; pairwise p-values 7.8e-6 for SD ~ PD, 2.7e-4 for OR ~ PD, and 7.0e-3 for SD ~ OR, Wilcoxon rank sum test). The fold change of CCF distinguishes the patient groups even better, which increases for PD, remains stable for SD, and decreases for OR patients (p-values 0.002, ~ 1, and 0.0001 respectively, Wilcoxon signed-rank test). Eleven drug-related mutations are identified from nine out of the 313 patients.

**Conclusions:**

The ctDNA fingerprint method improves both specificity and sensitivity of monitoring treatment response across several tumor types. It can identify tumor relapse/recurrence potentially earlier than imaging-based diagnosis. When augmented with tumor hotspot genes, it can track acquired drug-related mutations in patients.

## Background

Circulating tumor DNA (ctDNA) mainly stem from apoptotic or necrotic tumor cells released into the circulation system. They provide easily accessible snapshots of tumor burden and mutation profile and allow noninvasive longitudinal sampling [1–3]. These properties make ctDNA a promising tool to monitor tumor progression and treatment response [4–6]. Several studies have demonstrated that ctDNA had higher sensitivity and specificity than the traditional methods such as radiological imaging and blood protein biomarkers under some conditions [7, 8]. In metastatic colorectal cancer (CRC), the different patterns of ctDNA level change correlated with tumor responses in patients [9, 10]. Notably, there was no significant difference in the blood levels of common protein biomarker carcinoembryonic antigen (CEA) among different disease statuses in the study by Corcoran and Andre et al. [10]. In a triple-negative breast cancer cohort, all of the metastatic patients were found to be ctDNA-positive [11]. Encouraged by these results, clinic trials have been opened to test the clinic utility of ctDNA in the settings of pre-cancer, minimal residual disease, recurrent and/or metastatic disease, which are reviewed in Cescon et al. and Araujo et al. [6, 12].

Currently, the most prevalent way to analyze ctDNA employs next-generation sequencing (NGS) in combination with multiplex PCR to check a panel of hotspot mutations, which can be either generic to all tumors or specific to a certain cancer type [13, 14]. Although low cost and easy to design, ctDNA panels generated these ways can potentially miss important mutational clones in some patients if the mutations are not included in the panel targets, therefore result in inaccurate evaluation of disease status and treatment response. To address this problem, some researchers have tailored ctDNA panels to fit the genetic profiles of individual patients. In studying the clonal evolution and tumor relapse as part of the TRAcking non-small cell lung cancer evolution through therapy (TRACERx) study, Abbosh et al. used bespoke ctDNA panels to target clonal and sub-clonal single-nucleotide variants (SNVs) in patients [15]. The authors found that the tumor volume correlated with the variant allele frequency (VAF) and ctDNA monitoring could detect tumor recurrence earlier than clinical CT imaging. In another study, a ctDNA panel consisted of 9–24 founding mutations successfully detected residual disease in patients with breast cancer after adjuvant therapy [16].

In order to generalize the use of patient specific ctDNA panels across different tumor types and treatment regimens, and streamline the design process to make them accessible to individual clinical research units, we developed a new platform that is versatile and can be easily adapted to a range of clinical scenarios. We named the generated custom panels “ctDNA fingerprints” and applied it to longitudinal tracking of 313 Chinese patients who represented eight tumor types (colorectal carcinoma, hepatocellular carcinoma, lung cancer, cholangiocarcinoma, gastric carcinoma, breast cancer, pancreatic carcinoma, and esophageal carcinoma, ordered in case numbers) and had been exposed to seven categories of treatments (surgery, chemotherapy, radiotherapy, targeted drug therapy, immunotherapy, and combinations of them). Forty-five of these patients had matched clinical evaluation by imaging methods. We found the trend of ctDNA concentration change monitored by the ctDNA fingerprint panels faithfully tracked clinical outcomes. We also took the advantage of ctDNA monitoring to detect acquired drug-related mutations during tumor evolution with a pre-designed panel consisted of eight hotspot tumor genes. We found eleven drug-related mutations in total from 9 out of 313 patients monitored.

## Materials and methods

### Patients and study design

Three hundred and thirty-nine cancer patients were recruited from 20 hospitals in China between May 2016 and December 2018 and were followed up for a median of 6 months (range 1–16 months). All patients provided written, informed consent to use their genomic and clinical data for research purposes. All procedures involving human participants conformed to the ethical standards of the relevant institutions and/or national research committees, as well as the 1964 Helsinki Declaration and its subsequent amendments or similar ethical standards.

**Fig. 1:**
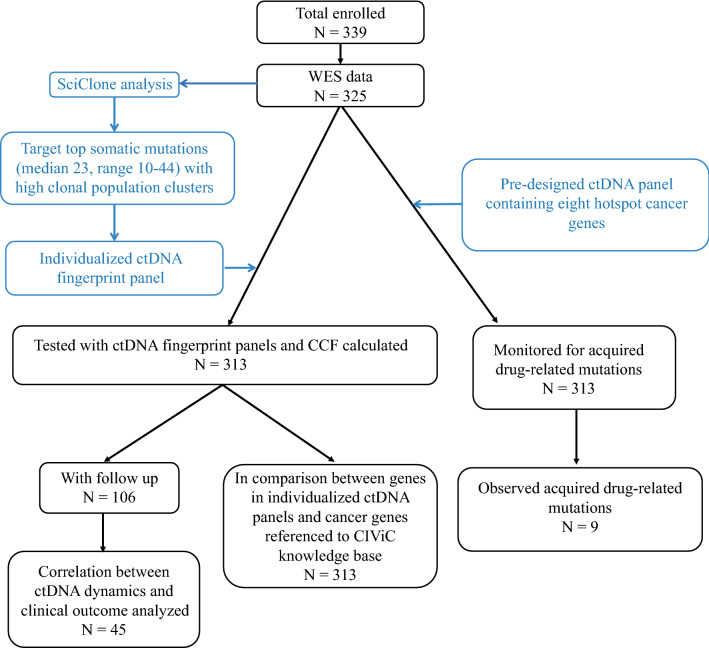
Flow chart of the study design. The black boxes represent the number and status of patients and the blue boxes represent the panel design

The study design is illustrated in Fig. 1. Whole exome sequencing (WES) data were successfully obtained from 325 patients, and ctDNA fingerprint panels, one panel for one patient, were designed and assessed. After excluding the patients with less than 10 mutations in their panels, 313 patients remained in the study. Follow-up information was available for 106 of the above patients. Among them, 45 patients received one clinical imaging diagnosis and 10 patients received two diagnoses, therefore 55 datasets are available for us to compare the performance of clinical imaging and the companion ctDNA monitoring. The treatment responses were evaluated by oncologists based on radiological images.

### Samples, DNA extraction and WES analysis

Tumor tissues (stored as FFPE blocks/slices) and patient matched germline control (peripheral whole blood) samples were collected. The tumor content in the tissue biopsies was assessed by pathologists. Only the samples with tumor content > 20% were used to ensure the sequencing and variant calling quality. Tumor DNA was extracted from FFPE slices using MagMAX FFPE DNA/RNA Ultra kit (cat# A31881, ThermoFisher), and germline DNA was extracted from the peripheral whole blood using Maxwell RSC blood DNA kit (cat# AS1400, Promega). DNA was sheared with a Covaris L220 sonicator and hybridized to the probes in Agilent SureSelect XT Human All Exon V5 kit (cat# 5990-9857EN, Agilent Technologies) for exome enrichment. Captured exome DNA was PCR amplified, end-repaired, attached to adapters and barcodes, and prepared to libraries using SureSelect XT HS and Low Input Library Preparation Kit for ILM (Pre PCR) kit (cat# G9507/9508 A through S, Agilent) according to the manufacturer’s instruction. The libraries were sequenced on an Illumina NovaSeq-6000 Sequencing System. Paired-end reads of 150 × 150 bp length were generated and the image analysis and base calling were done using Illumina onboard RTA3 program. After trimming adapters and removing low-quality reads, the reads were aligned to NCBI human genome reference assembly hg19 using the Burrows‐Wheeler Aligner (BWA) [17] alignment algorithm and further processed using the Genome Analysis Toolkit (GATK, version 3.5) [18], which includes the GATK Realigner Target Creator, to identify regions that needed to be realigned. Single-nucleotide variant (SNV), Indel, and copy number variation (CNV) were determined by using the MuTect/ANNOVAR/dbNSFP31, VarscanIndel, and CNVnator softwares respectively as reported in [19]. During the variant calling stage, the aligned sequences from the tumor sample were compared with the sequences from the peripheral whole blood from the same patient to generate a list of somatic mutations. The called somatic mutations were then filtered and annotated according to hg19 annotation using Variant Effect Predictor (VEP) [20]. The tumor mutational burden (TMB) is the total counts of nonsynonymous somatic mutations detected in WES as calculated using a published algorithm and reported as number of mutations per Mb in the study [21].

### ctDNA fingerprint panel

The individualized ctDNA fingerprint panels are designed to probe mainly somatic SNVs because they rely on multiplex PCR, which is prone to homopolymer-associated indel errors [22, 23]. First, clonal clusters were measured using SciClone tools [24] with the following parameters: tumor purity as determined by pathologists, and tumor copy number alterations (CNA), loss of heterozygosity (LOH) ratio in somatic mutation regions, and somatic variant allele frequency (VAF). The CNA and LOH were inferred from WES data using VarScan v2.4.2 tools [25]. Then, multiplex PCR primers specific to the top 10–45 somatic mutations within the high frequency clonal clusters were designed by Ion AmpliSeq Designer (ThermoFisher Scientific) and used in the ctDNA fingerprint panels. After filtering out amplicons of low amplification efficiency and inferior quality, the median number of somatic mutations in the assays was 23 (range 10–44).

### Hotspot tumor mutation panel

The pre-designed ctDNA panel contains eight common hotspot genes, namely BRAF, EGFR, ERBB2, KIT, KRAS, MET, NRAS, and PIK3CA [26, 27]. The specific mutations in these eight genes are listed in Additional file 1: Table S1.

### ctDNA isolation and detection

In principle, we collect blood for ctDNA isolation at 2 weeks and 4 weeks post the definitive treatment (if there was one), and in every 3 months after that. The actual schedule of a patient was set by his/her attending physician based on medical needs. 10 mL of whole blood was collected and stored in Streck’s BCT tubes (Streck, La Vista, USA). ctDNA in the plasma was extracted using MagMAX Cell-Free DNA Isolation kit (cat# 29319, ThermoFisher Scientific) and stored at − 20 °C until use. A minimum of 8 ng ctDNA was used as the template in the multiplex PCRs below. For the ctDNA fingerprint panels, DNA was amplified using KAPA2G Fast Multiplex Mix kit (cat# KK5802, KapaBiosystems, Wilmington, MA, USA), which has included barcode component, followed by Ion AmpliSeq Library kit 2.0 (cat# 4475345, ThermoFisher Scientific). For the pre-designed panel used for drug-related mutation detection, DNA was amplified using Ion AmpliSeq Library kit 2.0 in combination with barcoded adapters from Ion Xpress Barcode Adapters 1–16 kit (cat# 4471250, ThermoFisher Scientific). All libraries were sequenced using Ion Torrent S5 next-generation sequencing platform (ThermoFisher Scientific).

### CCF estimation

Raw reads from Ion Torrent S5 sequencing were filtered according to the following criteria: (1) > 70% bases had a quality score of Q20 and above; (2) > 40% reads mapped to the targeted template; (3) average coverage > 18,000 × ; and (4) > 80% amplicons had a coverage above 1000 × . The filtered reads were then aligned to human reference genome hg19 (Feb 2009 GRCh37/hg19) using BWA-0.7.12 [17], the data was formatted using Samtools-0.1.18 [28] and bedtools-2.17.0, and SNVs were identified with VarScan v2.4.2 [25] using the mpileup2snp program. The ctDNA content fraction (CCF) was estimated according to the following formula: $CCF = 2 \times \left( {\mathop \sum \limits_{i = 1}^{k} CTR_{i} } \right)/k$, in which *CTR*_*i*_ represents the fraction of the *i*-th somatic mutation allele in the ctDNA test and *k* represents the total number of mutations presented in the panel. Since ctDNA was monitored multiple times for each patient who may receive multiple clinical evaluations, we define the CCF sampled within 10 days of imaging examination or just before that as the definitive CCF, and the *fold change of CCF *=* (CCF*_*def − *_*CCF*_*pro*_*)/CCF*_*pro*_, in which *CCF*_*def*_ is the CCF of the definitive ctDNA test and *CCF*_*pro*_ is the CCF of the immediate proceeding ctDNA test.

### Reference samples

Reference samples used to evaluate the ctDNA detection threshold and performance were purchased from Horizon Discovery (Cambridge, UK), including Quantitative Multiplex Formalin Compromised (Mild) Reference Standard (cat# HD798), 1% Multiplex I cfDNA Reference Standard (cat# HD778), and 100% Wild-type (Tru-Q 0) (cat# HD752). The references were mixed and serially diluted to generate standards with allelic frequencies of 15%, 10%, 6%, 3%, 1%, 0.5%, 0.25%, and 0%.

### Analysis of fraction of genes and mutations with clinical interpretation

The clinical interpretation of genes and mutations was made using the Clinical Interpretation of Variants in Cancer (CIViC) knowledge base, an open source, community-driven web resource [29]. 414 genes and 3331 mutations were selected as reference objects because they have predictive, diagnostic, prognostic, and predisposing values and are predicted to be functional in cancers (Additional file 2: Table S2).

## Results

### Validation of the individualized ctDNA panels in reference samples

To determine the detection threshold and sensitivity of the ctDNA fingerprint panels, we performed more than 150 tests for each variant in 10 ng reference samples with total variant allelic frequencies at 15%, 10%, 6%, 3%, 1%, 0.5%, 0.25%, and 0% (See Materials and methods). The background ctDNA value in the wild-type reference was 0.065% ± 0.062% (mean ± S.D.), thus gave the upper limit of the background noise at 0.127%. Since each allele has two copies, the threshold CCF value for a positive detection was twice the reference sample threshold, approximately 0.25%. The specificity of detecting an individual mutation in the wild-type samples was 80.3%, and the sensitivities of the reference samples with expected frequencies of 0.1%, 0.25%, and 0.5% were 40.6%, 75.0%, and 96.3%, respectively. Mutations with a known frequency greater than 1% were all successfully detected (Table 1).

**Table 1:** Performance and threshold of the multiplex PCR ctDNA assay for single mutation detection

ctDNA concentration of standard	0%	0.10%	0.25%	0.50%	1.0%	3.0%	6.0%	10%	15%
Number of replicates	157	160	160	160	157	155	157	157	157
Observed allelic frequency									
Mean	0.065	0.122	0.276	0.577	1.132	3.380	6.358	9.858	15.2
Std. deviation	0.062	0.113	0.168	0.268	0.197	0.466	0.751	0.900	1.142
Std. error of mean	0.005	0.009	0.013	0.021	0.016	0.037	0.060	0.072	0.091
Lower 95% CI of mean	0.055	0.105	0.250	0.536	1.101	3.306	6.239	9.716	15.080
Upper 95% CI of mean	0.075	0.140	0.302	0.619	1.163	3.454	6.476	10.000	15.440
Minimum	0	0	0	0.020	0.660	2.100	4.740	7.660	11.690
25% percentile	0.010	0.030	0.123	0.373	1.010	3.050	5.800	9.290	14.310
Median	0.050	0.070	0.285	0.555	1.110	3.410	6.310	9.760	15.260
75% percentile	0.105	0.208	0.400	0.760	1.255	3.670	6.950	10.380	15.930
Maximum	0.230	0.410	0.760	1.430	1.770	4.940	8.200	14.430	18.490
Coefficient of variation	96.6%	92.2%	60.8%	46.4%	17.4%	13.8%	11.8%	9.1%	7.5%
Sensitivity (%, threshold = 0.127%)	NA	40.6%	75.0%	96.3%	100.0%	100.0%	100.0%	100.0%	100.0%

*NA* not applicable

In addition to the sensitivity of a single variant, we calculated that the sensitivities were 44.4% or 46.0%, 97.6% or 99.8%, and 99.99% or 100% for standard samples containing either five or ten mutants at concentrations of 0.10%, 0.25%, and 0.50%, respectively. The specificities were 98.7% and 99.9% for panels containing five and ten mutants, respectively.

Based on these results, patients with CCF values higher than 0.25% were considered to be ctDNA positive, although CCF values were still recorded for those lower than 0.25%. Moreover, the sensitivity and specificity were 99.99% and 99.9% for positive ctDNA detection when the panel had ten somatic SNVs.

### Application of ctDNA fingerprint panels and the pre-designed ctDNA panel

**Fig. 2:**
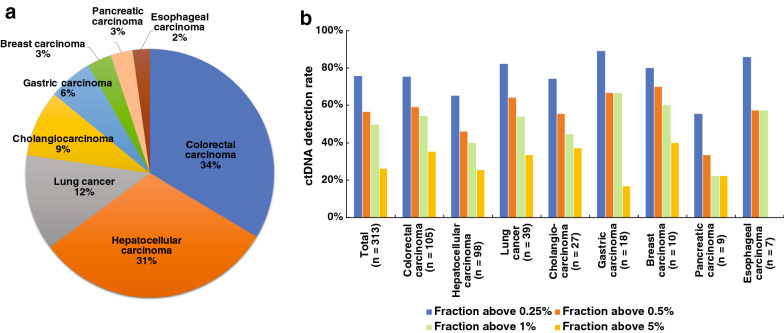
Overview of ctDNA tests. **a** Distribution of tumor types in 313 patients. **b** Fraction of patients with CCF values above 0.25%, 0.5%, 1%, and 5% grouped by cancer types

Our patient group includes eight different tumor types and the distribution is shown in Fig. 2a. CRC made up the highest proportion, followed by liver cancer, lung cancer, and cholangiocarcinoma in significant numbers. The other types included gastric, breast, pancreatic, and esophageal cancer. Three hundred and thirteen fingerprint panels, one for each patient, were designed. All patients underwent ctDNA tests for more than once, and some up to six times. In total, 888 individualized ctDNA tests were performed. We found that 75.9% of the patients were ctDNA positive, and 56.5%, 49.8%, and 26.3% of the patients had detectable ctDNA with a CCF value above 0.5%, 1%, and 5%, respectively (Fig. 2b).

We also monitored drug-related mutations using a pre-designed ctDNA panel. Out of the 313 patients and eight tumor types, we detected one potential acquired drug-related mutations in eight patients and three mutations in one patient (Additional file 3: Table S3). Six CRC patients acquired *KRAS* mutations with an allelic frequency > 0.05%. One lung adenocarcinoma patient acquired an *EGFR* p.T790M mutation, which was found 2 months before the imaging confirmed PD. The CCF value of the patient was 0.48% at the baseline, increased to 2.27% at the time of imaging, and decreased to 0.07% after 1 month on osimertinib treatment. *PIK3CA* mutations were detected in a cholangiocarcinoma patient and a breast cancer patient.

### ctDNA fingerprint panels are highly personalized

We analyzed the specificity of the ctDNA fingerprint panels. WES analysis detected 70,382 mutations in the 313 patients and 67,648 (96.1%) of them were detected only once. 7475 mutations were included in the ctDNA fingerprint panels. Of them, 6196 (96.0%) were detected in only one panel. On the other hand, 291 (93.0%) of the panels contained less than ten CIViC knowledge base curated, known cancer genes. Thus, these panels were highly specific to individual patients.

By comparing with the 414 tumor-associated genes and 3331 variants from the CIViC knowledge base (Additional file 2: Table S2), we calculated the occurrence of tumor-associated genes and variants in the ctDNA fingerprint panels. Most cancer-related genes and variants had a low incidence, with only 27 out of the 414 CIViC genes and 3 out of the 3331 variants occurred ten or more times in our cohort. The top ten frequently occurred genes and mutations are listed in Table 2. Moreover, 144 (46%) patients analyzed by the ctDNA fingerprint panels had 1–3 of these genes, 151 (47%) patients had 4–10 of these genes, and 10 (3%) patients did not contain the genes on the list. These results suggest that the ctDNA fingerprint panels may be used as low cost surrogate of WES to monitor changes in tumor-associated genes over time in patients and at the same time easily accessible because it requires only peripheral blood but not tumor tissue.

**Table 2:** Top ten most frequently occurred genes and mutations in our cohort that are curated by CIViC knowledge base

Gene	Number of detections	Frequency of detection %	Mutation	Number of detections	Frequency of detection %
*TP53*	192	61.3	*KRAS* p.G12D	28	8.95
*APC*	79	25.2	*KRAS* p.G13D	13	4.15
*KRAS*	72	23.0	*TP53* p.R249S	10	3.19
*EGFR*	34	10.9	*EGFR* p.L858R	9	2.88
*PIK3CA*	31	9.90	*KRAS* p.G12V	9	2.88
*SMAD4*	27	8.63	*KRAS* p.G12C	9	2.88
*ARID1A*	26	8.31	*TP53* p.R175H	9	2.88
*CTNNB1*	22	7.03	*BRAF* p.V600E	6	1.92
*GNAQ*	18	5.75	*TP53* p.R273C	6	1.92
*KMT2C*	18	5.75	*TP53* p.R273H	6	1.92

### Clinical outcomes and the level and dynamics of ctDNA

Forty-five patients and 55 paired datasets of clinical evaluation and ctDNA monitoring (10 patients received two imaging evaluations) were available which included 15 PD, 26 SD and 14 OR cases. These patients had different cancers and had received various treatment regimens, including operations, chemotherapy, radiotherapy, targeted therapy, immunotherapy, multiple combination treatment, and liver transplantation (Additional file 4: Table S4), thus representing a wide spectrum of cases that might be encountered at clinics. The median and mean intervals between the definitive and its proceeding ctDNA tests were 2 and 2.4 months, respectively. The definitive ctDNA test was defined to be the sampling within 10 days or just before the clinical imaging evaluation. The longest interval was 8 months in a patient with hepatocellular carcinoma who showed good imaging response 4 months after the first ctDNA test, so the patient did not undergo the second (definitive) ctDNA test until 8 months later. Thirty-five definitive ctDNA tests were done in the same month as the imaging examination, and 25 definitive ctDNA tests were done 1–3 months before the imaging evaluation. This gave us the chance to analyze the correlation of ctDNA measurements with treatment responses and disease status in a variety of clinical scenarios in different cancers.

**Fig. 3:**
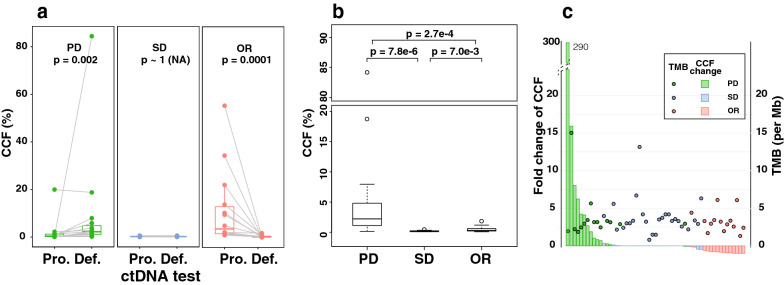
Level and change of ctDNA concentration in patients with clinical outcome data. **a** Paired CCF comparison of the definitive (Def.) and proceeding (Pro.) ctDNA tests of individual cases. The p-values are calculated by the Wilcoxon singed-rank test. Additionally, the null hypothesis of no difference among the groups is tested by the Kruskal–Wallis test and it gives a p-value of 1.36e-6. **b** Boxplot of CCF of the definitive ctDNA test. The pairwise p-values are calculated by the Wilcoxon rank sum test. **c** Waterfall plot of fold change of CCF and the matched TMB from the initial WES. Bars, fold change of CCF (left axis). Filled circles, TMB as counts per Mb sequences monitored (right axis). Additionally, the null hypothesis of no difference among the groups is tested by the Kruskal–Wallis test and it gives a p-value of 5.64e-10. PD: progressive disease, 15 cases; SD: stable disease, 26 cases; OR: objective response, 14 cases

The change of CCF over time was significant for the patients with PD and OR (p-value = 0.002 and 0.0001 respectively, Wilcoxon signed-rank test) but not significant for patients with SD (p-value approaching 1 due to tied and zero values, Wilcoxon signed-rank test) (Fig. 3a).

We defined the ctDNA test closest in time to the clinical evaluation by imaging as the definitive test. The distribution of CCF of the definitive ctDNA test among the three disease status groups was not the same, which is supported by the Kruskal–Wallis test (p-value = 1.36e-6). The CCF values of the SD and OR patients were one order of magnitude lower than that of the PD patients (Fig. 3b). The median and mean CCF values of the definitive ctDNA test for patients with PD, SD and OR were 2.22% and 8.96%, 0.18% and 0.21%, and 0.31% and 0.54%, respectively. The corresponding pairwise p-values are 7.8e-6 for SD ~ PD, 2.7e-4 for OR ~ PD, and 7.0e-3 for SD ~ OR by Wilcoxon rank sum test (Figure 3b).

The dynamics of individualized ctDNA can also be characterized by fold change of CCF between the definitive and its proceeding ctDNA tests (Fig. 3c). Overall, the difference of fold change of CCF among different patient groups is significant (p-value = 5.64e-10 by Kruskal–Wallis test). The 15 PD cases showed a ctDNA positive status in their definitive ctDNA test, and the median fold change of CCF was ~ 2 (Fig. 3). The maximum fold change of CCF up to 290 was observed in a patient with brain metastases of breast cancer. The CCF of the patient was 2.26% pre-operation then dropped substantially to 0.29% after surgical removal of the brain metastases. The patient experienced metastasis and relapsed 3 months after that, during which time the CCF value increased again to 84.4%. We also found that the CCF values of two of the PD patients were decreased rather than increased, which were 0.2% vs. 0.16% and 19.95% vs. 18.76% in the proceeding and definitive ctDNA tests. The median CCF value of the fourteen OR cases decreased 86.3%. The CCF of 11 of the OR cases changed from above 1% to below 1%. Furthermore, five of the OR cases had no detectable ctDNA (CCF < 0.25%) at the definitive test. Eighteen of the SD cases showed no detectable ctDNA at both time points, and two patients showed no fluctuation between the two tests. In total, 73% SD patients had no change in their CCF values (Fig. 3 and Additional file 4: Table S4).

These results indicated that either the dynamics of ctDNA or the absolute value of CCF at the definitive ctDNA test is highly correlated with clinical outcome although the readout of the dynamics can distinguish clinical outcomes more easily. The fold change of CCF would increase for PD, remain stable for SD, and decrease for OR patients. As a comparison, TMB from WES was calculated but we found no correlation between TMB and clinical outcomes or fold change of CCF (Fig. 3c).

### Dynamics of each mutation in ctDNA fingerprint panels

**Fig. 4:**
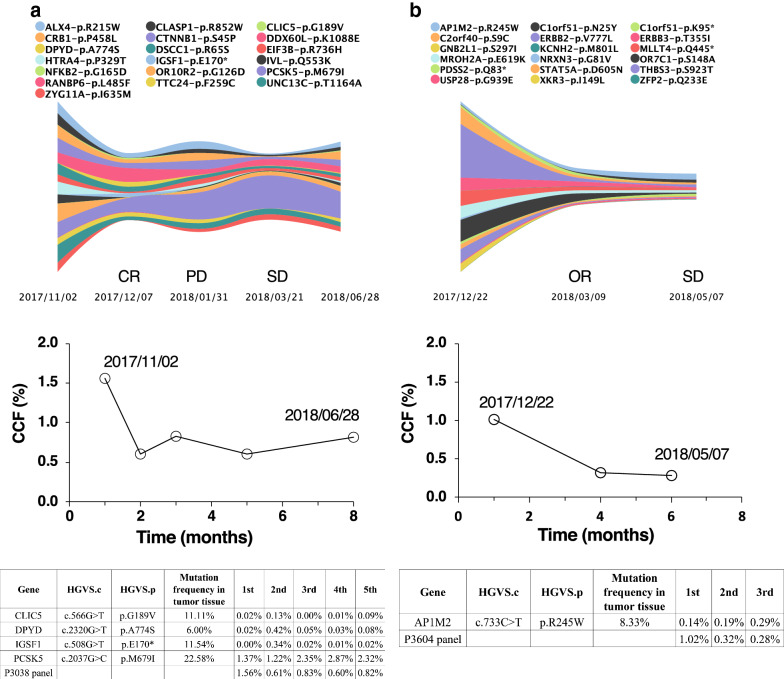
Examples of dynamics of individual mutations and their sum detected by personalized ctDNA panels. **a** A hepatocellular carcinoma patient; **b** A breast cancer patient. Top row, stream plot representing the change of individual mutations. The colors indicate different genes and specific mutations on the panels; Middle row, overall change (sum) of all mutations on the panels; Bottom row, tables highlight the dynamics of single mutations that were inconsistent with the overall trend

Two patients had three clinical evaluations. This made it possible for us to analyze the changes of mutation composition and the frequency of each mutation over time and treatment regimens by using the ctDNA fingerprint panel (Fig. 4). Patient A (male, hepatocellular carcinoma) received an operation on October 23, 2017 and used liver protecting drugs after the operation. CR was achieved at the beginning of December. However, bone metastasis occurred only 1 month later. Radiotherapy was then applied, and the patient’s condition was controlled. The patient participated in an immunotherapy clinical trial in May 2018. The ctDNA levels of *DPYD* and *IGSF1* increased significantly at the second detection (12/07/2017); however, the overall ctDNA content, as represented by CCF, decreased, and the patient achieved CR in clinical evaluation. *PCSK5* increased slightly at the fourth ctDNA detection (03/21/2018), but the overall ctDNA content of the panel decreased, corresponding with the clinical status of SD. Patient B (female, breast cancer) was treated with chemotherapy. The concentration of *AP1M2* was below 0.25% in both ctDNA tests, but the CCF value changed from 1.02 to 0.32%, consistent with an OR status of this patient by clinical evaluation. These results indicate that the fluctuations in individual mutations may differ from the overall trend of the panel and clinical evaluation. Therefore, it is necessary to follow a panel of patient specific somatic mutations in ctDNA analysis. Following only one or a few mutations, as most droplet PCR based platforms do, may increase the risk of misdiagnosis.

## Discussion

Although proof-of-concept studies have confirmed the clinical value of ctDNA in predicting some cancer patients’ response to treatment [30], a panel of experts from the American Society of Clinical Oncology and the College of American Pathologists concluded that most of the clinical validity and practicability of ctDNA testing reported to date are insufficient [31]. Several challenges must be overcome before ctDNA analysis can be translated into clinical practice. Toward this end, we developed a strategy to design patient-specific ctDNA panels with high sensitivity and specificity, which can be applied to patients with multiple tumor types and several different treatments.

Our customized panels achieved a sensitivity of 99.99% when 10 SNVs were used in the assessment and the detection threshold was set at 0.25% of the total circulating DNA for individual SNVs. A median of 23 SNVs were detected in our customized panels, so the effective sensitivity was higher. On the other hand, the specificity of the panels was 99.93% in the reference samples. The ctDNA detection rate was ~ 76% in all patients, and ~ 80% in patients with lung cancer, gastric, breast, and esophageal cancer. In comparison, a previous in-depth sequencing study of more than 70 tumor-associated genes in more than 20,000 patients with solid tumors showed a ctDNA detection rate of 85% overall and a detection rate of 90% in patients with liver cancer, non-small cell lung cancer, and gastric cancer [32].

Previous studies showed that ctDNA level was an early marker for treatment response in CRC patients who received surgery or chemotherapy and ctDNA monitoring could detect recurrence earlier than radiographic imaging [33–35]. Monitoring with a panel of 61 hotspot genes revealed that an increase of ctDNA level was associated with clinical PD in patients with muscle-invasive bladder cancer [36]. In the present study, we retrospectively collected data from patients with multiple cancer types who were treated with multiple treatment options. We showed that either the absolute value definitive CCF or fold change of CCF can clearly distinguish PD patients from SD and OR patients (Fig. 3), thus confirmed the diagnostic value of our customized ctDNA panels in more expanded cancer types.

More importantly, when the changes in ctDNA levels were compared with corresponding imaging evaluation, the results of the two approaches were highly consistent, suggesting the potential clinical validity of our panels. After treatment, the CCF values of the OR patients all decreased, and increased in 13 out of 15 (87%) PD patients. The CCF values of 21 out of 26 (81%) SD patients showed no change; the change in CCF values of the remaining SD patients was from − 45.65 to 32.56%. The CCF value slightly reduced (from 19.95 to 18.76% in mutant fraction) in a patient with cholangiocarcinoma, but the absolute value was still exceedingly high, indicating the treatment failed to contain the tumor. Indeed, the patient had a PD status in clinical evaluation. Nevertheless, there were exceptions. The ctDNA levels and treatment response in patients with pancreatic cancer were not as consistent as in the other cancers we tested. A PD patient with pancreatic cancer had their CCF value below the detection threshold but we don’t know the cause.

We also noticed that the dynamics of a single mutation can be inconsistent with the overall trend of the mutations in a panel (Fig. 4). In a patient with hepatocellular carcinoma who achieved CR status, the CCF value decreased, whereas *DPYD* and *IGSF1* increased significantly. In another patient with breast cancer, *AP1M2* was below 0.25% in consecutive tests, but the patient achieved OR and her CCF value changed from 1.02 to 0.32%. Our findings corroborate the literature. In a breast cancer patient whose status changed from SD to PD, the ctDNA content of most mutations increased, but the level of *ZFYVE21*, a known breast cancer marker, continued to decrease [37]. Similarly, the level of *NRAS*, a known melanoma marker, was reduced by tenfold in a melanoma patient, but the patient’s disease nevertheless progressed and so did the CCF value of the patient [38]. Therefore, monitoring the changes in single mutations, even for those associated with known cancer related genes, is insufficient for predicting clinical outcome and can sometimes reach a wrong conclusion. It is important to monitor multiple patient-specific mutations simultaneously, just as our customized panels and the associated CCF calculation are designed for, to avoid potential misdiagnosis.

As reported, somatic cell-free DNA consists of variants derived from both tumor cells and clonal expansion of hematopoietic cells; thus, it is hard to judge whether a mutation is derived from the tumor or hematopoietic sources [39, 40]. Although ultra-deep sequencing can solve this issue, it is very expensive to do and is limited to known hotspots. This is one of the major motivations for us to use paired WES data from patient tumors and blood to screen the variants specific to the tumor and free from the interference from the clonal hematopoietic cells. Additionally, the ctDNA fingerprint panels displayed high specificity for all patients because they are based on WES data of individual patients. 95.6% of mutations were detected only once across the board. Also, 93.0% of individualized ctDNA panels harbored less than ten known tumor genes curated by the CIViC knowledge base. These properties make our custom designed panels highly specific to each individual patient. A median of 23 patient-specific mutations, corresponding to ~ 4-kb sequences, were included in the panels. This made the cost of sequencing and analysis of the ctDNA fingerprint panels much lower than reported ctDNA panels which targeted > 50 tumor hotspot genes [41, 42].

In order to gain insights about the development of drug resistance during cancer evolution, as well as provide information of eligibility to newly available targeted drugs, we tested a pre-designed ctDNA panel with eight hotspot cancer genes. Among the 313 patients, we identified 11 drug-related mutations in 9 patients. Those includes *EGFR* c.2369C > T p.T790M, an important biomarker for patients with lung cancer. It generally appears after patients develop resistance to first-line EGFR inhibitors, and those patients with *EGFR* p.T790M mutation can obtain clinical benefits by treatment with osimertinib.

Nevertheless, the ctDNA fingerprint assay presented above has some limitations. It requires prior WES analysis of the tumor tissue before ctDNA monitoring, which is difficult or impossible for some patients. Furthermore, this assay only targets SNVs because short sequencing length is used, therefore changes of indels and longer range variants cannot be detected, including gene fusions/chromosomal rearrangements and copy number variations (CNVs), which are highly valuable diagnostic tools for some tumors [43, 44].

Tumor mutational burden has been confirmed to be an important biomarker because its association with patient survival and response to immune checkpoint inhibition therapy [45, 46]. An effective way to track patient TMB during treatment and disease progression would provide valuable reference for patients and tending physicians. The design of ctDNA fingerprint panel uses WES, so patient TMB at the time of panel design can be calculated from the WES data (Fig. 3c). We found no clear correlation of TMB from the time of ctDNA fingerprint panel design to clinical outcomes, nor to CCF value and fold change. The fingerprint panels contain only up to 44 probes which are variable (by definition) from patient to patient, so they are not suitable to track patient TMB change over time. Dynamic monitoring of TMB is better to be done with a fixed panel with a large number of probes, for example, FoundationOne CDx (Foundation Medicine), QIAseq TMB panel (Qiagen) or similar commercial products, or even better with ctDNA analysis through whole-exome capturing and sequencing. However, these methods are either lack of specificity to patients or potentially bear higher cost.

Another shortcoming of our study is the number of patients in some cancer types was small. This makes the conclusion in these cancer types less robust then other studies with more patients, for example, non-small cell lung cancer [47] and CRC [48]. Cautions need to be applied when interpreting data from these minor groups of cancers, and future study with more patients is warranted.

While this manuscript is in preparation, Reinert et al. and Wang et al. published their studies using customized ctDNA panels in colorectal cancer patients to detect relapse/recurrence and tumor response to adjuvant chemotherapy [48, 49]. Consistent to our findings, the two studies found serial measurement of ctDNA could detect recurrence with a higher sensitivity than carcinoembryonic antigen (CEA) level and also with a time earlier than routine radiographic imaging, and hinted that ctDNA can be used to stratify patients for postoperative management.

## Conclusions

We developed a new ctDNA platform that can successfully monitor treatment responses in a variety of cancer types for patients who are exposed to multiple treatments. Moreover, a combination of individualized panels and panels with acquired drug-related mutations can more comprehensively monitor the dynamics of ctDNA and tumor clone evolution for prompt treatment decisions.

## Supplementary information

**Additional file 1: Table S1.** Mutations of eight hotspot tumor genes in the pre-designed ctDNA panel.

**Additional file 2: Table S2.** 414 genes and 3331 variants from CIViC knowledge base (02-mar-2020 version).

**Additional file 3: Table S3.** Acquired drug-related mutations in nine patients identified using a pre-designed ctDNA panel.

**Additional file 4: Table S4.** 55 paired data sets for CCF in two consecutive ctDNA detections, treatment regimens, and imaging evaluations.

## Data Availability

The dataset used and analyzed and source code used in this study are available from the corresponding authors upon reasonable request. The raw genetic materials and data are under the regulation of Chinese government and cannot be shared to any organization or private person outside of China in any way.
